# 3-Ammonio-4-hydroxy­benzoate monohydrate

**DOI:** 10.1107/S1600536809017462

**Published:** 2009-05-14

**Authors:** Sami Ullah, M. Nawaz Tahir, Durre Shahwar, Zaheer-ud-Din Khan, Muhammad Akmal Khan

**Affiliations:** a Department of Chemistry, Government College University, Lahore, Pakistan; b Department of Physics, University of Sargodha, Sargodha, Pakistan; c Department of Botany, Government College University, Lahore, Pakistan

## Abstract

The title compound, C_7_H_7_NO_3_·H_2_O, which crystallized as a hydrate, was obtained from an extraction of the plant species *Saussurea atkinsonii* of the asteraceae family collected from the hilly area (Ayubia) of Pakistan during the flowering season. The dihedral angle between the benzene ring and the carboxyl­ate group is 25.64 (5)°. In the crystal, the packing is consolidated by N—H⋯O and O—H⋯O hydrogen bonds, as well as weak aromatic π–π stacking [centroid–centroid separation = 3.9365 (9) Å] and C=O⋯π inter­actions.

## Related literature

For a related structure, see: Bertasso *et al.* (2001 ▶). For reference structural data, see: Allen *et al.* (1987 ▶).
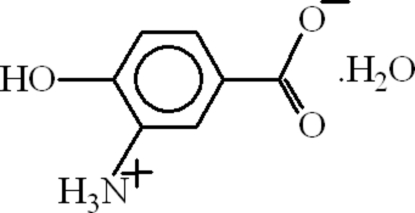


## Experimental

### 

#### Crystal data

C_7_H_7_NO_3_·H_2_O
*M* *_r_* = 171.15
Orthorhombic, 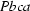

*a* = 8.7711 (3) Å
*b* = 12.7193 (7) Å
*c* = 12.9289 (6) Å
*V* = 1442.38 (11) Å^3^
*Z* = 8
Mo *K*α radiation
μ = 0.13 mm^−1^
*T* = 296 K
0.26 × 0.20 × 0.20 mm

#### Data collection

Bruker Kappa APEXII CCD diffractometer
Absorption correction: multi-scan (*SADABS*; Bruker, 2005 ▶) *T*_min_ = 0.971, *T*_max_ = 0.976
8827 measured reflections
1725 independent reflections
1277 reflections with *I* > 2σ(*I*)
*R*_int_ = 0.033

#### Refinement

*R*[*F* ^2^ > 2σ(*F* ^2^)] = 0.042
*wR*(*F* ^2^) = 0.118
*S* = 1.06
1725 reflections
127 parameters
H atoms treated by a mixture of independent and constrained refinement
Δρ_max_ = 0.31 e Å^−3^
Δρ_min_ = −0.24 e Å^−3^

### 

Data collection: *APEX2* (Bruker, 2007 ▶); cell refinement: *SAINT* (Bruker, 2007 ▶); data reduction: *SAINT*; program(s) used to solve structure: *SHELXS97* (Sheldrick, 2008 ▶); program(s) used to refine structure: *SHELXL97* (Sheldrick, 2008 ▶); molecular graphics: *ORTEP-3 for Windows* (Farrugia, 1997 ▶) and *PLATON* (Spek, 2009 ▶); software used to prepare material for publication: *WinGX* (Farrugia, 1999 ▶) and *PLATON*.

## Supplementary Material

Crystal structure: contains datablocks global, I. DOI: 10.1107/S1600536809017462/hb2969sup1.cif

Structure factors: contains datablocks I. DOI: 10.1107/S1600536809017462/hb2969Isup2.hkl

Additional supplementary materials:  crystallographic information; 3D view; checkCIF report

## Figures and Tables

**Table 1:** Hydrogen-bond geometry (Å, °)

*D*—H⋯*A*	*D*—H	H⋯*A*	*D*⋯*A*	*D*—H⋯*A*
N1—H1*A*⋯O1^i^	0.952 (18)	1.945 (18)	2.8884 (16)	170.4 (14)
N1—H1*A*⋯O2^i^	0.952 (18)	2.335 (18)	2.9008 (18)	117.6 (13)
N1—H1*B*⋯O4^ii^	0.944 (19)	2.001 (19)	2.8957 (19)	157.4 (16)
N1—H1*C*⋯O1^iii^	0.933 (17)	1.860 (17)	2.7846 (18)	170.5 (16)
O3—H3⋯O4^iv^	0.904 (18)	1.760 (18)	2.6456 (15)	166.0 (19)
O4—H41⋯O2^iv^	0.90 (2)	1.80 (2)	2.6945 (18)	171.1 (17)
O4—H42⋯O1^iii^	0.884 (19)	2.03 (2)	2.9027 (18)	168.8 (17)
C6—H6⋯O3^v^	0.93	2.55	3.446 (2)	161
C7—O2⋯CgA^vi^	1.25 (1)	3.49 (1)	3.9313 (16)	101 (1)

Symmetry codes: (i) 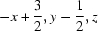; (ii) 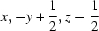; (iii) 
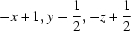
; (iv) 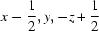; (v) 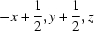; (vi) 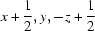.

**Fig. 1.**
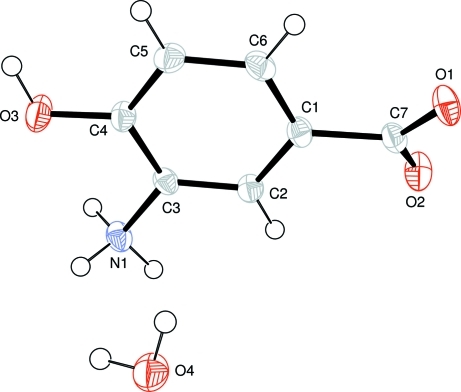
View of (I) with displacement ellipsoids drawn at the 50% probability level. H-atoms are shown by small circles of arbitrary radius.

**Fig. 2.**
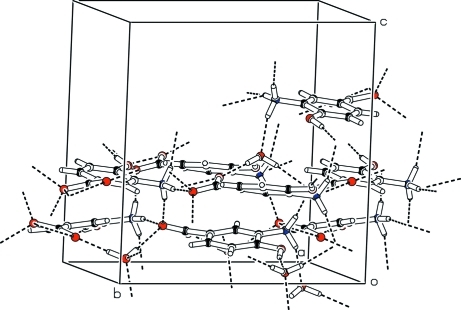
The partial packing of (I), showing that molecules form three-dimensional polymeric network.

## supplementary crystallographic information

### Comment

The medicinal plants are available all over the world. Locally available plant specie Saussurea atkinsonii of asteraceae family was collected from hilly area (Ayubia) of Pakistan during the flowering season. The plant was dried inside room for 20–25 days. The title compound (I), (Fig. 1), is an extract of it in chloroform and methanol. The study of its bio-activity is in progress.

The crystal structure of (II) (4-vinylphenyl 3-amino-4-hydroxybenzoate or bagremycin A (Bertasso *et al.*, 2001), has been reported which contains the aromatic ring along with heavy atoms of the substituants of (I). In the title compound, the bond distances and bond angles are within normal ranges (Allen *et al.*, 1987). The benzene ring A (C1–C6) is planar and is oriented at a dihedral angle of 25.64 (5)° with the CO_2_ group. The N-atom of ammonium is in plane of the ring A, whereas the O-atom of hydroxy group is at a distance of -0.0580 (21) Å from the same.

There exist intensive intermolecular H-bonding (Table 1), resulting in three-dimensional polymeric network. There also exist CgA···CgA^i^ [symmetry code i = 1 - *x*, 1 - *y*, -*z*] interaction at a distance of 3.9365 (9) Å, where CgA is the centroid of aromatic ring. The molecules may also be stabilized due to C==O···π interaction (Table 1).

### Experimental

The Specie Saussurea atkinsonii of asteraceae family was dried inside room for 20–25 days as a whole and grinded. The extract was obtained using soxhlet apparatus in 50% chloroform and 50% methanol and it was subjected to isolation by performing column chromatography and thin layer chromatography. The extract obtained was recrystallized from methanol and light brown rods of (I) were obtained. The water found in the structure was presumably incorporated from the atomsphere.

### Refinement

The coordinates of H-atoms of hydroxy, ammonium moiety and water molecule were refined. The other H-atoms were positioned geometrically, with C-H = 0.93 Å for aromatic type and constrained to ride on their parent atoms, with U_iso_(H) = 1.2U_eq_(C, N, O).

### Crystal data

**Table:**

C_7_H_7_NO_3_·H_2_O	*F*(000) = 720
*M**_r_* = 171.15	*D*_x_ = 1.576 Mg m^−^^3^
Orthorhombic, *P**b**c**a*	Mo *K*α radiation, λ = 0.71073 Å
Hall symbol: -P 2ac 2ab	Cell parameters from 1725 reflections
*a* = 8.7711 (3) Å	θ = 3.2–28.3°
*b* = 12.7193 (7) Å	µ = 0.13 mm^−^^1^
*c* = 12.9289 (6) Å	*T* = 296 K
*V* = 1442.38 (11) Å^3^	Rod, light brown
*Z* = 8	0.26 × 0.20 × 0.20 mm

### Data collection

**Table:**

Bruker Kappa APEXII CCD diffractometer	1752 independent reflections
Radiation source: fine-focus sealed tube	1277 reflections with *I* > 2σ(*I*)
graphite	*R*_int_ = 0.033
Detector resolution: 7.40 pixels mm^-1^	θ_max_ = 28.3°, θ_min_ = 3.2°
ω scans	*h* = −11→11
Absorption correction: multi-scan (*SADABS*; Bruker, 2005)	*k* = −15→16
*T*_min_ = 0.971, *T*_max_ = 0.976	*l* = −11→17
8827 measured reflections	

### Refinement

**Table:**

Refinement on *F*^2^	Primary atom site location: structure-invariant direct methods
Least-squares matrix: full	Secondary atom site location: difference Fourier map
*R*[*F*^2^ > 2σ(*F*^2^)] = 0.042	Hydrogen site location: inferred from neighbouring sites
*wR*(*F*^2^) = 0.118	H atoms treated by a mixture of independent and constrained refinement
*S* = 1.06	*w* = 1/[σ^2^(*F*_o_^2^) + (0.0684*P*)^2^ + 0.0409*P*] where *P* = (*F*_o_^2^ + 2*F*_c_^2^)/3
1725 reflections	(Δ/σ)_max_ < 0.001
127 parameters	Δρ_max_ = 0.31 e Å^−^^3^
0 restraints	Δρ_min_ = −0.24 e Å^−^^3^

### Special details

**Table:**

Geometry. Bond distances, angles *etc*. have been calculated using the rounded fractional coordinates. All su's are estimated from the variances of the (full) variance-covariance matrix. The cell e.s.d.'s are taken into account in the estimation of distances, angles and torsion angles
Refinement. Refinement of *F*^2^ against ALL reflections. The weighted *R*-factor *wR* and goodness of fit *S* are based on *F*^2^, conventional *R*-factors *R* are based on *F*, with *F* set to zero for negative *F*^2^. The threshold expression of *F*^2^ > σ(*F*^2^) is used only for calculating *R*-factors(gt) *etc*. and is not relevant to the choice of reflections for refinement. *R*-factors based on *F*^2^ are statistically about twice as large as those based on *F*, and *R*- factors based on ALL data will be even larger.

### Fractional atomic coordinates and isotropic or equivalent isotropic displacement parameters (Å^2^)

**Table:**

	*x*	*y*	*z*	*U*_iso_*/*U*_eq_	
O1	0.69598 (12)	0.67023 (9)	0.16442 (9)	0.0284 (4)	
O2	0.83764 (11)	0.53206 (10)	0.12269 (9)	0.0302 (4)	
O3	0.20823 (11)	0.29862 (10)	0.11448 (10)	0.0319 (4)	
N1	0.48231 (14)	0.21872 (11)	0.16388 (11)	0.0232 (4)	
C1	0.57236 (15)	0.50443 (12)	0.14203 (11)	0.0205 (5)	
C2	0.58869 (15)	0.39732 (13)	0.15731 (11)	0.0203 (4)	
C3	0.46511 (14)	0.33137 (12)	0.14912 (11)	0.0193 (4)	
C4	0.32078 (15)	0.37051 (12)	0.12444 (12)	0.0212 (4)	
C5	0.30300 (15)	0.47766 (13)	0.11230 (12)	0.0241 (5)	
C6	0.42742 (15)	0.54444 (13)	0.12153 (12)	0.0232 (4)	
C7	0.71208 (15)	0.57284 (13)	0.14299 (11)	0.0216 (5)	
O4	0.43081 (12)	0.34489 (11)	0.45169 (10)	0.0308 (4)	
H1A	0.587 (2)	0.2008 (14)	0.1721 (12)	0.0278*	
H1B	0.4519 (19)	0.1828 (14)	0.1034 (15)	0.0278*	
H1C	0.4229 (18)	0.1948 (14)	0.2189 (14)	0.0278*	
H2	0.68403	0.36974	0.17325	0.0243*	
H3	0.119 (2)	0.3252 (16)	0.0913 (15)	0.0383*	
H5	0.20714	0.50524	0.09785	0.0289*	
H6	0.41402	0.61656	0.11398	0.0279*	
H41	0.3898 (19)	0.4057 (16)	0.4289 (15)	0.0369*	
H42	0.381 (2)	0.2927 (16)	0.4217 (15)	0.0369*	

### Atomic displacement parameters (Å^2^)

**Table:**

	*U*^11^	*U*^22^	*U*^33^	*U*^12^	*U*^13^	*U*^23^
O1	0.0316 (5)	0.0154 (7)	0.0383 (7)	−0.0051 (4)	0.0053 (4)	−0.0044 (5)
O2	0.0231 (5)	0.0245 (7)	0.0430 (7)	−0.0032 (5)	0.0043 (4)	−0.0063 (5)
O3	0.0193 (5)	0.0217 (7)	0.0548 (8)	−0.0032 (4)	−0.0059 (5)	0.0000 (6)
N1	0.0214 (6)	0.0150 (8)	0.0331 (8)	0.0010 (5)	0.0027 (5)	0.0015 (6)
C1	0.0236 (7)	0.0164 (9)	0.0216 (8)	−0.0029 (5)	0.0013 (5)	−0.0026 (6)
C2	0.0183 (6)	0.0190 (9)	0.0235 (8)	0.0006 (5)	0.0011 (5)	−0.0006 (6)
C3	0.0209 (6)	0.0142 (9)	0.0228 (8)	0.0003 (5)	0.0018 (5)	0.0013 (6)
C4	0.0195 (6)	0.0188 (9)	0.0254 (8)	−0.0024 (5)	−0.0004 (5)	−0.0019 (6)
C5	0.0205 (6)	0.0218 (9)	0.0300 (9)	0.0041 (6)	−0.0024 (6)	0.0003 (7)
C6	0.0290 (7)	0.0137 (8)	0.0270 (8)	0.0014 (6)	0.0000 (5)	0.0005 (7)
C7	0.0265 (7)	0.0169 (9)	0.0214 (8)	−0.0044 (6)	0.0013 (5)	−0.0002 (6)
O4	0.0257 (5)	0.0255 (8)	0.0412 (7)	0.0017 (5)	−0.0044 (4)	0.0023 (6)

### Geometric parameters (Å, °)

**Table:**

O1—C7	1.277 (2)	C1—C7	1.503 (2)
O2—C7	1.2453 (17)	C1—C6	1.3948 (19)
O3—C4	1.3518 (18)	C1—C2	1.384 (2)
O3—H3	0.904 (18)	C2—C3	1.375 (2)
O4—H41	0.90 (2)	C3—C4	1.3972 (19)
O4—H42	0.884 (19)	C4—C5	1.381 (2)
N1—C3	1.453 (2)	C5—C6	1.388 (2)
N1—H1C	0.933 (17)	C2—H2	0.9300
N1—H1A	0.952 (18)	C5—H5	0.9300
N1—H1B	0.944 (19)	C6—H6	0.9300
			
C4—O3—H3	114.3 (13)	O3—C4—C5	125.06 (12)
H41—O4—H42	107.7 (17)	C3—C4—C5	118.69 (13)
C3—N1—H1A	110.5 (11)	O3—C4—C3	116.26 (13)
C3—N1—H1B	109.8 (11)	C4—C5—C6	120.36 (13)
H1A—N1—H1B	104.4 (14)	C1—C6—C5	120.64 (15)
H1A—N1—H1C	112.1 (14)	O1—C7—O2	123.20 (14)
C3—N1—H1C	111.3 (11)	O2—C7—C1	118.57 (14)
H1B—N1—H1C	108.4 (15)	O1—C7—C1	118.24 (12)
C2—C1—C6	118.72 (13)	C3—C2—H2	120.00
C2—C1—C7	118.97 (12)	C1—C2—H2	120.00
C6—C1—C7	122.24 (14)	C4—C5—H5	120.00
C1—C2—C3	120.53 (13)	C6—C5—H5	120.00
N1—C3—C2	120.64 (12)	C1—C6—H6	120.00
C2—C3—C4	120.97 (14)	C5—C6—H6	120.00
N1—C3—C4	118.38 (12)		
			
C6—C1—C2—C3	2.1 (2)	C1—C2—C3—C4	0.5 (2)
C7—C1—C2—C3	−175.02 (13)	N1—C3—C4—O3	−1.1 (2)
C2—C1—C6—C5	−2.7 (2)	N1—C3—C4—C5	178.75 (14)
C7—C1—C6—C5	174.30 (14)	C2—C3—C4—O3	177.60 (14)
C2—C1—C7—O1	−156.35 (14)	C2—C3—C4—C5	−2.5 (2)
C2—C1—C7—O2	23.8 (2)	O3—C4—C5—C6	−178.25 (15)
C6—C1—C7—O1	26.7 (2)	C3—C4—C5—C6	1.9 (2)
C6—C1—C7—O2	−153.17 (15)	C4—C5—C6—C1	0.7 (2)
C1—C2—C3—N1	179.24 (13)		

### Hydrogen-bond geometry (Å, °)

**Table:**

*D*—H···*A*	*D*—H	H···*A*	*D*···*A*	*D*—H···*A*
N1—H1A···O1^i^	0.952 (18)	1.945 (18)	2.8884 (16)	170.4 (14)
N1—H1A···O2^i^	0.952 (18)	2.335 (18)	2.9008 (18)	117.6 (13)
N1—H1B···O4^ii^	0.944 (19)	2.001 (19)	2.8957 (19)	157.4 (16)
N1—H1C···O1^iii^	0.933 (17)	1.860 (17)	2.7846 (18)	170.5 (16)
O3—H3···O4^iv^	0.904 (18)	1.760 (18)	2.6456 (15)	166.0 (19)
O4—H41···O2^iv^	0.90 (2)	1.80 (2)	2.6945 (18)	171.1 (17)
O4—H42···O1^iii^	0.884 (19)	2.03 (2)	2.9027 (18)	168.8 (17)
C6—H6···O3^v^	0.93	2.55	3.446 (2)	161
C7—O2···CgA^vi^	1.2453 (17)	3.4940 (13)	3.9313 (16)	101.24 (9)

Symmetry codes: (i) −*x*+3/2, *y*−1/2, *z*; (ii) *x*, −*y*+1/2, *z*−1/2; (iii) −*x*+1, *y*−1/2, −*z*+1/2; (iv) *x*−1/2, *y*, −*z*+1/2; (v) −*x*+1/2, *y*+1/2, *z*; (vi) *x*+1/2, *y*, −*z*+1/2.
